# Utility of extracellular vesicles in the diagnosis of central nervous system diseases

**DOI:** 10.3389/fneur.2026.1959745

**Published:** 2026-10-19

**Authors:** Minghao Wang, Junwen Yang, Conghao Gao, Siyu Qu, Hongyu Mi, Yu Guo, Ting Gui, Zhibo Gai

**Affiliations:** 1 School of Medicine, Shandong University of Traditional Chinese Medicine, Jinan, Shandong, China; 2 College of Traditional Chinese Medicine, Shandong University of Traditional Chinese Medicine, Jinan, Shandong, China; 3 Medical School of Ophthalmology and Optometry, Shandong University of Traditional Chinese Medicine, Jinan, Shandong, China; 4 School of Acupuncture-Moxibustion and Tuina, Shandong University of Traditional Chinese Medicine, Jinan, Shandong, China; 5 Experimental Center, Shandong University of Traditional Chinese Medicine, Jinan, China; 6 Key Laboratory of Traditional Chinese Medicine Classical Theory, Ministry of Education, Shandong University of Traditional Chinese Medicine, Jinan, China

**Keywords:** Alzheimer’s disease, biomarkers, central nervous system diseases, early diagnosis, extracellular vesicles, Parkinson’s disease

## Abstract

Central nervous system (CNS) disorders encompass a diverse spectrum of conditions characterized by intricate pathological mechanisms, and the presence of the blood–brain barrier (BBB) has long impeded early diagnosis. Extracellular vesicles (EVs) are lipid bilayer nanoparticles released by living cells that can encapsulate and transport bioactive molecules such as proteins and nucleic acids across the BBB. EVs can be detected in biofluids such as blood and cerebrospinal fluid (CSF) through non-invasive or minimally invasive assays, and have emerged as highly promising vehicles for CNS liquid biopsies. Recent studies demonstrate that specific EV-associated protein cargoes (e.g., phosphorylated tau, α-syn) and non-coding RNAs (e.g., miR-422a, miR-409-3p) exhibit distinct differential expression profiles across various pathologies. These molecular signatures hold promise for early detection, differential diagnosis, and disease staging, with some biomarker panels showing high diagnostic accuracy. However, whether they outperform conventional CSF biomarkers still requires direct comparison and validation within the same cohorts. This review highlights recent research advances in the use of EVs for the diagnosis of Alzheimer’s disease and Parkinson’s disease, briefly touches upon other CNS disorders including stroke, glioma, multiple sclerosis, Huntington’s disease, and amyotrophic lateral sclerosis, and discusses the clinical translational potential and remaining challenges of EV-based diagnostics.

## 1 Introduction

Central nervous system (CNS) diseases encompass a wide variety of conditions, including neurodegenerative diseases characterized by progressive neuronal loss and functional decline, such as Alzheimer’s and Parkinson’s diseases; acute brain injury like stroke; and immune-mediated or neoplastic conditions, including multiple sclerosis and gliomas. Nearly one-sixth of the world’s population suffers from CNS diseases (1) Owing to their complex pathophysiology and the restrictive nature of the blood–brain barrier (BBB), early diagnosis of CNS diseases remains a significant challenge (2). Current clinical diagnostics rely heavily on neuroimaging and surgical biopsy; however, these diagnostic modalities are either insensitive to early-stage molecular alterations or highly invasive, rendering them unsuitable for mass screening (3). While liquid biopsy has emerged as a promising alternative, the BBB severely restricts the efflux of brain-derived biomarkers into the bloodstream, diluting peripheral concentrations to ultra-low levels and compromising diagnostic sensitivity (4). Conversely, cerebrospinal fluid (CSF) directly mirrors the biochemical milieu of the brain—as exemplified by well-established biomarkers like p-Tau and Aβ42 in Alzheimer’s disease diagnosis—but its collection requires a lumbar puncture. This invasive procedure is associated with low patient acceptance and practical limitations in clinical practice (3). To circumvent these limitations, EVs have attracted significant attention as emerging vehicles for intercellular communication. Because they encapsulate molecular information in the CNS, cross the blood–brain barrier, and remain stable within peripheral circulation, EVs hold the potential to offer a transformative paradigm for CNS liquid biopsies (5, 6). Building on this premise, the enrichment of specific brain-derived EVs holds immense potential for non-invasive stratification of disease stages in intracranial pathological processes (Figure 1).

**Figure 1:**
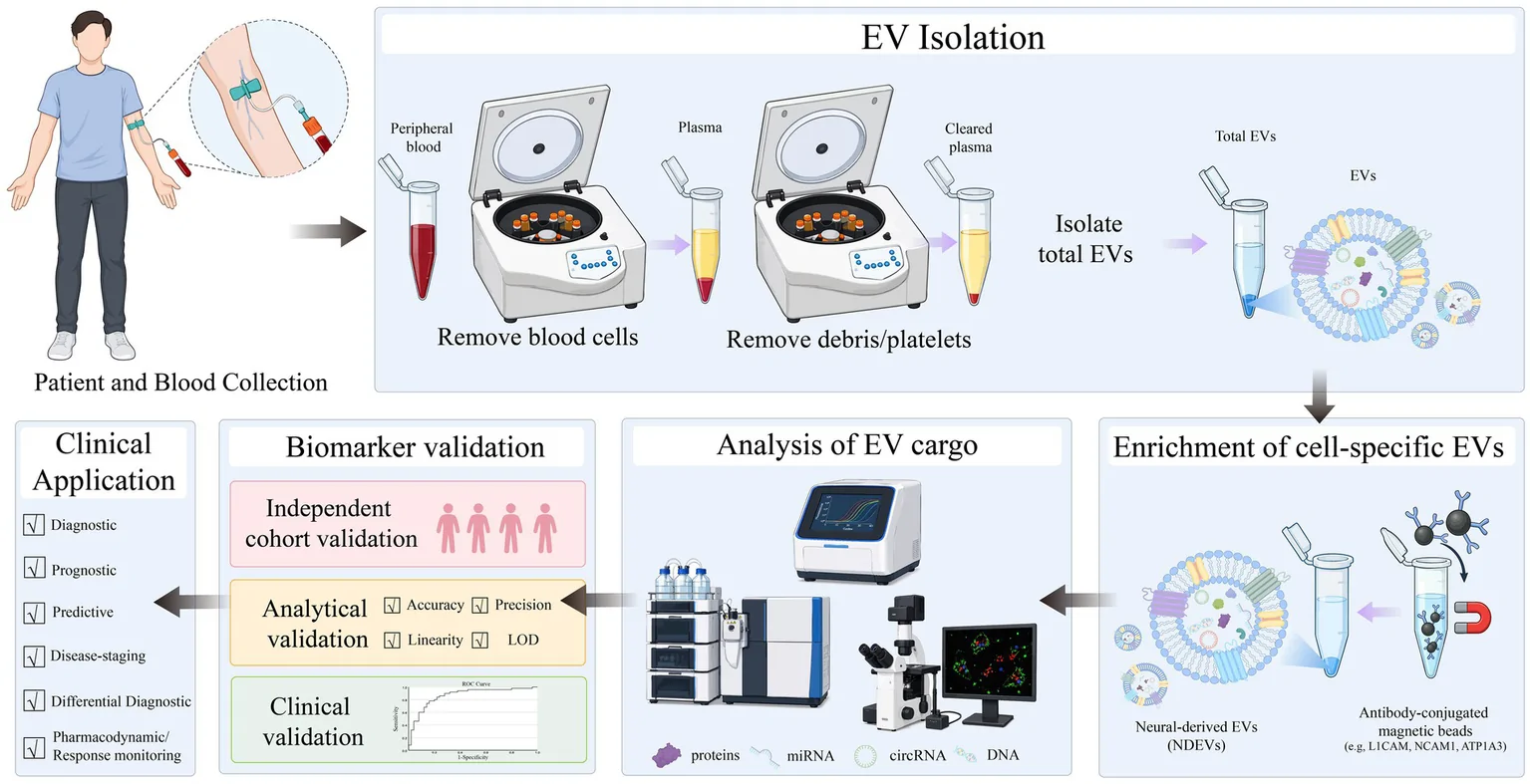
Overview of the translational workflow for EV-based biomarkers in CNS diseases. Peripheral blood is collected from patients, followed by differential centrifugation to remove blood cells, debris/platelets, and to isolate total EVs. Cell-specific EV enrichment, including putative neuron-derived EVs (NDEVs), using antibody-conjugated magnetic beads targeting candidate markers such as L1CAM, NCAM1, or ATP1A3. The EV cargo, including proteins, miRNAs, circRNAs, and DNA, is analyzed using multiple analytical platforms. Candidate biomarkers then undergo analytical validation (accuracy, precision, linearity, LOD) and independent cohort validation, followed by clinical validation using ROC analysis. Validated EV biomarkers can ultimately be applied in clinical settings for diagnosis, prognosis, prediction, disease staging, differential diagnosis, and pharmacodynamic/response monitoring.

This narrative review searched PubMed, Web of Science, and Embase from database inception to January 2026. English-language literature was searched using the following keywords: (“Extracellular Vesicles”[Mesh] OR “Exosomes”[Mesh] OR exosomal[tiab] OR “neuron-derived extracellular vesicles”[tiab] OR NDEVs[tiab])AND(“Alzheimer Disease”[Mesh] OR “Parkinson Disease”[Mesh] OR “Huntington Disease”[Mesh] OR “Stroke”[Mesh] OR “Multiple Sclerosis”[Mesh] OR “Amyotrophic Lateral Sclerosis”[Mesh] OR “Glioma”[Mesh])AND(biomarker[ti] OR diagnosis[ti] OR diagnostic[ti])AND(blood[tiab] OR plasma[tiab] OR serum[tiab] OR “cerebrospinal fluid”[tiab]). Studies were considered eligible if they investigated extracellular vesicle-associated molecular cargo in human biofluids (blood, serum, plasma, or cerebrospinal fluid) for the diagnosis, differential diagnosis, prognosis, disease staging, or monitoring of central nervous system diseases. Studies reporting biomarker performance metrics, including area under the receiver operating characteristic curve (AUC), sensitivity, specificity, or longitudinal clinical associations, were preferentially included and discussed. Studies based solely on cellular or animal models without validation in human biofluids were not considered primary clinical biomarker evidence. Studies that reported only differential expression without evaluating biomarker diagnostic performance, or that used non-fluid human samples such as brain tissue or fibroblasts, were regarded only as mechanistic or supportive evidence. Studies using non-fluid human samples, such as brain tissue-derived EVs or fibroblasts, were considered mechanistic or supportive evidence when they provided relevant information on disease-associated EV cargoes or biological mechanisms. Studies focusing primarily on engineered or modified EVs for therapeutic delivery, drug loading, vaccine development, or EV-based therapeutic applications were excluded, unless they provided methodological insights or supporting evidence relevant to EV-based diagnostics. An initial search yielded 152 records; after removal of duplicates and screening, approximately 26 studies were ultimately included as primary clinical biomarker evidence, with approximately 15 additional studies regarded as mechanistic or supportive evidence. As this is a narrative review, no formal risk-of-bias assessment or meta-analysis was performed.

## 2 Biogenesis and biological characteristics of extracellular vesicles

Extracellular vesicles (EVs) are lipid bilayer-delimited particles released by cells. According to MISEV2023, the generic term “EVs” should be used unless a specific biogenesis pathway has been experimentally demonstrated. Operationally, EVs may be described according to size as small EVs (sEVs; typically <200 nm) or large EVs (lEVs; typically >200 nm), although these boundaries are method-dependent and do not imply a specific cellular origin. Biogenesis-based terms, such as “exosomes” for multivesicular body (MVB)-derived EVs or “ectosomes/microvesicles” for plasma membrane-derived EVs, should therefore be reserved for cases in which their specific origin has been experimentally established (7). Among EV cargoes, proteins, lipids, and non-coding RNAs are selectively enriched, conferring a tissue-of-origin molecular signature that reflects the parental cells, including CNS residents such as neurons, microglia, and astrocytes (4). The clinical potential of EVs is driven by several advantages: their lipid bilayer confers stability and protects cargo from enzymatic degradation, permitting long-term biobanking (8).; they can be isolated from accessible biofluids such as blood, urine, and saliva (9); and their membranes facilitate biomembrane fusion, enabling them to traverse biological barriers, most notably the BBB (10). In the context of CNS disorders, immunocapture targeting L1CAM has been widely investigated as an operational strategy to enrich putative neuron-derived EVs (NDEVs) from peripheral blood. However, L1CAM is a putative rather than definitive neuronal EV marker, and its specificity and biological validity remain debated. A detailed critical appraisal of L1CAM-based enrichment, alternative neuronal markers, and cell-of-origin verification strategies is provided in the Discussion (11). Collectively, the stability, barrier-permeating capacity, and tissue-of-origin signatures of EVs offer a promising analytical platform for the early diagnosis of CNS disorders. Although EVs can cross the BBB and enter the circulation, blood EVs are predominantly derived from haematopoietic cells, endothelial cells, and other peripheral sources (12). CNS-derived EVs are estimated to constitute only a minor fraction of total plasma EVs, and their signals may be further diluted by EVs from blood cells and other peripheral tissues (12). Consequently, when a circulating EV-associated molecule is proposed as a CNS biomarker, it is essential to consider whether it truly originates from the CNS or reflects peripheral contributions. This caveat applies to all blood-based EV biomarkers discussed below, and should be taken into account when interpreting their diagnostic value (Figure 2).

**Figure 2:**
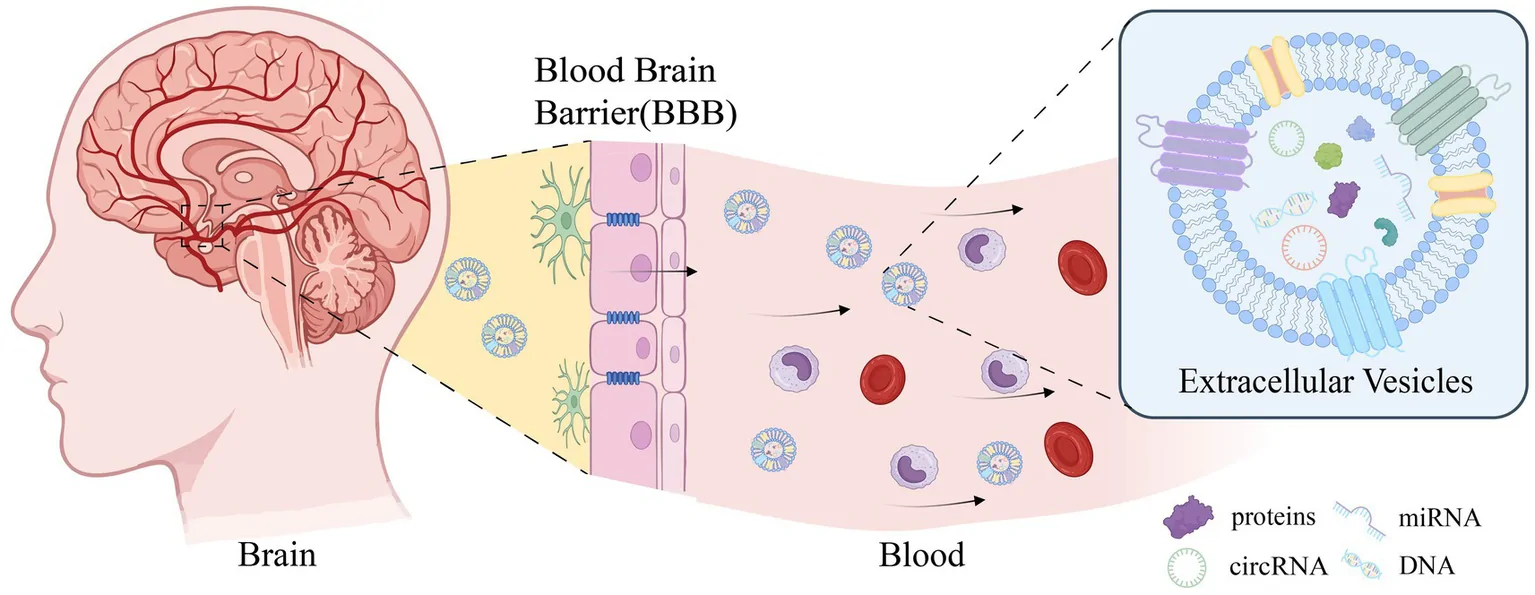
Neuron-derived extracellular vesicles crossing the Blood–Brain Barrier. EVs released by neurons and other brain cells encapsulate bioactive molecules, including proteins, miRNAs, circRNAs, and DNA, within a lipid bilayer. These EVs can traverse the blood–brain barrier (BBB) and enter peripheral blood, thereby transferring molecular information from the central nervous system to the circulation. The protein, miRNA, circRNA, and DNA cargoes carried by EVs reflect the physiological or pathological state of their parental cells, highlighting their potential as liquid biopsy biomarkers for central nervous system diseases, including early diagnosis and disease staging. The inset on the right illustrates the typical structure of an EV and its cargoes, including proteins, miRNAs, circRNAs, and DNA.

## 3 Extracellular vesicles as biomarkers in Alzheimer’s disease

As a neurodegenerative disease, Alzheimer’s disease (AD) is pathologically characterized by extracellular amyloid- beta (Aβ) plaques and the accumulation of intracellular neurofibrillary tangles (NFTs) composed of phosphorylated tau protein (p-tau). Crucially, these pathological cascades initiate 15 to 20 years prior to the manifestation of clinical symptoms, highlighting the vital necessity of biomarker discovery for early diagnosis (13–15).

Kapogiannis et al. demonstrated that, during the preclinical phase of AD, neuron-derived extracellular vesicles (NDEVs) from individuals who subsequently developed AD exhibited increased levels of p-tau181 and p-tau231, accompanied by elevated phosphorylated IRS-1 and increased nEV diameter, whereas total tau levels remained comparable with those of controls. These longitudinal divergences suggest that phosphorylated tau species, particularly when integrated with other nEV-derived alterations, may provide improved predictive performance for early AD detection (14). Complementing these findings, Jia et al. demonstrated that Aβ42, t-tau, and p-tau181 levels in peripheral immunoenriched EVs are significantly elevated in patients with AD compared with individuals with amnestic mild cognitive impairment (aMCI). Crucially, the diagnostic efficacy of these plasma EV biomarkers is comparable to that of CSF biomarkers (16). Although NDEV enrichment partially mitigates the concern that p-tau can also be released from peripheral tissues, whether these plasma EV signals truly reflect CNS-derived vesicles remains to be fully established. Fiandaca et al. noted that whereas p-tau181, p-tau396, and Aβ42in extracts of blood NDEVs are elevated up to a decade prior to clinical onset, p-tau396 remains at baseline in patients with frontotemporal dementia (FTD), suggesting that p-tau396 may serve as a specific differential marker to distinguish AD from FTD (17).

Beyond these classical proteomic markers, synaptic proteins detected in plasma NDEVs—including synaptophysin, synaptopodin, synaptotagmin-2, and neurogranin—are significantly decreased in both AD and FTD. Conversely, growth-associated protein 43 (GAP43) and synapsin 1 exhibit AD-selective reductions, whereas their alterations differ in FTD, with GAP43 showing no significant change and total synapsin 1 displaying a paradoxical increase compared with controls. Intriguingly, levels of several NDEV synaptic proteins, particularly synaptopodin and synaptophysin, correlate with cognitive scores, whereas conventional neurotoxic proteins (Aβ42 and p-tau181) show no such correlation. These findings suggest that NDEV synaptic proteins may provide complementary information to conventional AD biomarkers and may have potential as indicators of disease progression (18). Furthermore, neuroinflammatory and homeostatic dysregulation can be monitored via astrocyte-derived EVs (ADEVs), wherein complement regulatory proteins (such as CD59) exhibit a preclinical decline, offering an early diagnostic window (19). Concurrently, intra-vesicular elevations of cathepsin D, lysosome-associated membrane protein 1 (LAMP-1), and ubiquitinylated proteins, paired with a suppression of heat-shock protein 70 (HSP70) within blood NDEVs, highlight a systemic endolysosomal dysfunction that could serve as an early diagnostic index (20). Su et al. found that brain-derived EVs (BDEVs) show elevated plasmalogen ethanolamines (e.g., PE(P-36:2)) and an approximately 50% reduction in DHA-containing lipids such as LPE(22:6), suggesting AD-associated lipid remodeling (21).

Notably, miR-212 is significantly downregulated in blood-derived NDEVs from AD patients and exhibits considerable diagnostic potential (22). To overcome the sensitivity limitations of single analytes, multiplexed assays may improve diagnostic performance. In one study, a serum-derived small extracellular vesicle (sEV)-associated miRNA panel combining miR-135a, −193b, and −384 achieved high diagnostic efficacy for early-stage AD (MCI), supporting the potential of multiplexed profiling to enhance diagnostic sensitivity (23). Furthermore, EV-associated miRNA profiles may reflect region-specific pathological changes in AD. Devara et al. reported that miR-501-3p and miR-502-3p levels in EVs were elevated in both CSF and serum from patients with AD, with their levels positively correlated with the density of amyloid plaques and neurofibrillary tangles in the entorhinal cortex, amygdala, and hippocampus (Table 1) (24).

**Table 1:** Fluid and tissue-derived biomarkers for the diagnosis, prognosis, and prediction of AD.

Biomarker/Panel	Source	EV population/Type	Change	Control group	AUC	Study design	Significance	Reference
Multiplexed nEV model (p-tau181, p-tau231, p-IRS-1, nEV diameter, plus other nEV measures)	Blood	NDEVs	Increase (p-tau181, p-tau231, p-IRS-1, nEV diameter)	Cognitively normal controls	Training: 0.896 (95% CI, 84.5–94.8; *n* = 161); Test: 0.800 (95% CI, 63.9–90.7; *n* = 80)	Retrospective study	Predictive	(14)
Aβ42, T-tau and p-tau181 panel	Blood	immunoenriched EVs	Increase (in AD vs. aMCI and controls)	Healthy controls; aMCI	Validation set: Combined 0.980 (AD vs. control; *n* = 73 AD, 72 controls), 0.880 (AD vs. aMCI; *n* = 73 AD, 71 aMCI), 0.85 (aMCI vs. control; *n* = 71 aMCI, 72 controls); individual AUCs 0.72–0.93; 95% CI not reported	Prospective study	Diagnostic (for AD and aMCI)	(16)
P-S396-tau	Blood	NDEVs	Increase	Healthy controls	–	Retrospective study	Differential diagnosis (AD vs. FTD)	(17)
Aβ42, p-tau181and P-S396-tau panel	Blood	NDEVs	Increase	Healthy controls; FTD	AD vs. control: 0.999 (*n* = 57 AD, 57 control); FTD vs. controls: 0.992 (P-T181-tau; *n* = 16 FTD, 16 FTC); individual AUCs 0.731–0.991	Retrospective study	Diagnostic	
Cathepsin D, ubiquitinylated protein, and HSP70 panel	Blood	NDEVs	Increase (Cathepsin D)	Cognitively normal controls;	AD vs. control: cathepsin D AUC 1.0 (*n* = 26 AD, 26 controls); composite model AUC 1.0 (*n* = 26 AD, 26 AC);	Retrospective study	Diagnostic	(20)
			Increase (ubiquitinylated protein)			Retrospective study		
			Decrease (HSP70)			Retrospective study		
CD59	Plasma	ADEVs	Decrease	Healthy controls	–	Retrospective study	Diagnostic	(19)
GAP43	Plasma	NDEVs	Decrease	Healthy controls	–	Retrospective study	Differential diagnosis (AD vs. FTD)	(18)
miR-135a, miR-384 and miR-193b panel	Serum	sEVs	Increase (miR-135a)	Healthy controls	For MCI diagnosis: combination AUC 0.995 (95% CI, 0.973–1.000); *n* = 101 MCI, 228 controls	Prospective study	Diagnostic (for early-stage AD (MCI))	(23)
			Increase (miR-384)			Prospective study		
			Decrease (miR-193b)			Prospective study		
miR-212	Plasma	NDEVs	Decrease	Cognitively intact biomarker-negative controls (NCI)	AD vs. NCI: AUC 0.84 (95% CI, 0.72–0.96; *n* = 16 AD, 31 NCI)	Retrospective study	Diagnostic	(22)
miR-501-3p and miR-502-3p panel	CSF and serum	EVs	Increase	Healthy controls	combined: UC vs. AD AUC 0.916 (*n* = 25 AD, 19 UC);	Retrospective study	Diagnostic	(24)

AUC, area under the curve; CI, confidence interval; *n*, number of participants/samples; AD, Alzheimer’s disease; aMCI, amnestic mild cognitive impairment; FTD, frontotemporal dementia; FTC, frontotemporal dementia control; MCI, mild cognitive impairment; NCI, no cognitive impairment; UC, unaffected controls; NDEVs, neuron-derived extracellular vesicles; ADEVs, astrocyte-derived extracellular vesicles; BDEVs, brain-derived extracellular vesicles; sEVs, small extracellular vesicles.

## 4 Extracellular vesicles as biomarkers in Parkinson’s disease

As the second most prevalent neurodegenerative condition, Parkinson’s disease (PD) poses a severe clinical threat to the aging population (25). The histopathological hallmark of PD is the aberrant intracellular aggregation of α-synuclein (α-syn) (26), a pathological cascade that directly underscores the diagnostic utility of EV-associated biomolecules.

Jiang et al. demonstrated that α-synuclein levels in serum neuron-derived EVs from patients with PD were approximately two-fold higher than those in healthy controls, achieving an AUC of 0.86 for distinguishing clinical PD from controls. Notably, α-synuclein levels were also elevated in individuals with rapid eye movement sleep behavior disorder, suggesting their potential relevance in the prodromal phase of PD. Furthermore, a combined biomarker panel incorporating α-synuclein and clusterin improved differential diagnostic performance, distinguishing PD from other proteinopathies, including frontotemporal dementia, progressive supranuclear palsy, and corticobasal syndrome, as well as from multiple system atrophy (27). A subsequent meta-analysis by Kim et al. validated that total α-syn levels are significantly elevated in both total EVs and NDEVs from the plasma of PD cohorts, supporting the potential utility of sEV-associated α-syn as a liquid biopsy biomarker for PD (28). Beyond cross-sectional diagnosis and differential segregation, sEV-associated signatures may serve as objective indices of clinical severity. Kitamura et al. isolated plasma sEVs from PD patients staged at Hoehn and Yahr (HY) grades II and III. Utilizing two-dimensional difference gel electrophoresis (2D-DIGE), they identified a significant depletion of clusterin, complement C1r subcomponent, and apolipoprotein A1 (ApoA1) compared to healthy controls. Notably, peripheral sEV-associated ApoA1 levels were markedly lower in HY stage III compared to stage II, displaying a strong negative correlation with disease severity (*r* < −0.77, *p* < 0.01). These findings suggest that sEV-associated ApoA1 may reflect disease severity and serve as a potential disease-staging biomarker in PD (29).

In central nervous system compartments, Gui et al. revealed that miRNA profiles in CSF-derived sEVs differentiate PD patients from healthy controls, identifying 16 upregulated and 11 downregulated miRNAs. Among these, miR-409-3p alone achieved high diagnostic accuracy for PD discrimination, while its combination with miR-153 further improved diagnostic performance (30). Similarly, Cristina T et al. established a robust diagnostic model by integrating CSF-derived EV-associated miR-10b-5p, miR-22-3p, miR-151a-3p, and total α-syn levels, yielding strong diagnostic accuracy (31). Paralleling these central alterations, systemic variations are also detectable in peripheral circulation. Cao et al. validated the downregulation of miR-19b alongside the upregulation of miR-24 and miR-195 in total serum EVs. Integration of these three miRNAs into a unified serum panel demonstrated high diagnostic accuracy for PD diagnosis (32). To map these transcriptomic shifts directly to clinical staging, He et al. leveraged Weighted Gene Co-expression Network Analysis (WGCNA) on serum EV-derived miRNA sequencing datasets, cross-validating candidates via Receiver Operating Characteristic (ROC) curves and quantitative real-time PCR (qRT-PCR). This bioinformatic framework revealed a specialized miRNA panel tracking with definitive Hoehn-Yahr (H-Y) stages: miR-374a-5p and miR-374b-5p are universally upregulated across all active phases (stages II–IV); miR-199a-3p undergoes a stage-specific downregulation restricted to stage II; miR-28-5p is selectively upregulated in stage III; and miR-22-5p and miR-151a-5p display divergent upregulation and downregulation, respectively, at stage IV. These stage-dependent dynamics underscore the expansive horizons of EV-associated miRNAs for non-invasive screening and disease staging (33). Building on these findings, miR-24 has been reported to be upregulated in both serum and CSF-derived EVs, and miR-331-5p is consistently elevated across CSF, serum, and plasma EVs, emerging as a promising cross-fluid candidate for PD. These observations support further exploration of EV-associated miRNAs in peripheral liquid biopsy (Table 2) (34).

**Table 2:** Fluid and tissue-derived biomarkers for the diagnosis, prognosis, and prediction of PD.

Biomarker/panel	source	EV population/type	change	Control group	AUC	Study design	Significance	Reference
Total α-syn	Blood	NDEVs	Increased	Healthy controls	–	Secondary study	Diagnostic	(28)
α-syn	Serum	NDEVs	Increased (2-fold in PD, PDD, DLB, and prodromal RBD vs. controls; not elevated in MSA or other proteinopathies)	Healthy controls; MSA and other proteinopathies as disease controls	PD vs. HC: 0.860 (training group *n* = 314: PD 201, HC 113; validation group *n* = 105: PD 74, HC 31); sensitivity 0.82–0.85, specificity 0.71–0.74	Prospective study	Diagnostic	(27)
α-syn	Serum	NDEVs	Increased in prodromal RBD (similar to PD levels); remains stably elevated over 40.3 ± 8.5 months	Prodromal RBD	–	Prospective study	Predictive	
α-syn and clusterin panel	Serum	NDEVs	Ratio (α-syn/clu) increased	Other proteinopathies (FTD/PSP/CBS)	0.980	Prospective study	Differential diagnosis	
			Increased	MSA	0.940	Prospective study	Differential diagnosis	
Clusterin	Plasma	sEVs	Decreased	Healthy controls	–	Prospective study	Diagnostic	(29)
complement C1r subcomponent		sEVs	Decreased	Healthy controls	–	Prospective study	Diagnostic	
ApoA1		sEVs	Decreased	Healthy controls	–	Prospective study	Disease-staging (correlated with disease severity)	
miR-153 and miR-409-3p panel	CSF	sEVs	Increased	Healthy controls	combination: 0.990 (95% CI 0.934–1.090); validation cohort *n* = 78 PD, 35 HC	Prospective study	Diagnostic	(30)
α-syn, miR-22-3p, miR-10b-5p and miR-151a-3p panel	CSF	EVs	Decreased (α-syn)	Healthy controls	0.960 (*n* = 40 early-stage PD, 40 controls)	Retrospective study	Diagnostic	(31)
			Decreased (miR-22-3p)					
			Increased (miR-10b-5p)					
			Increased (miR-151a-3p)					
miR-19b, miR-24 and miR-195 panel	Serum	EVs	Decreased (miR-19b)	Healthy controls	0.946 (95% CI, 0.910–0.981; *n* = 109 PD, 40 controls)	Retrospective study	Diagnostic	(32)
			Increased (miR-24)					
			Increased (miR-195)					
hsa-miR-374a-5p	Serum	sEVs	Increased (in stages II, III, and IV)	Healthy controls	Stage II vs. control: 0.758 (95% CI 0.5721–0.994); Stage III vs. control: 0.780 (95% CI 0.6733–0.8858); Stage IV vs. control: 0.763 (95% CI 0.6213–0.9036); *n* = 103	Prospective study	Diagnostic, differentiating each stage of PD from healthy controls	(33)
hsa-miR-374b-5p	Serum	sEVs	Increased (in stages II, III, and IV)	Healthy controls	Stage II vs. control: 0.798 (95% CI 0.6084–0.9884); Stage III vs. control: 0.742 (95% CI 0.6240–0.8598); Stage IV vs. control: 0.761 (95% CI 0.6182–0.9038); *n* = 103	Prospective study	Diagnostic, differentiating each stage of PD from healthy controls	
hsa-miR-199a-3p	Serum	sEVs	Decreased (specifically Decreased d in stage II)	Healthy controls	Stage II vs. control: 0.738 (95% CI 0.5597–0.9161); Stage II vs. stage III: 0.729 (95% CI 0.5493–0.9091); Stage II vs. stage IV: 0.756 (95% CI 0.5597–0.9517); *n* = 103	Prospective study	Disease-staging (stage II-specific)	
hsa-miR-28-5p	Serum	sEVs	Increased (specifically Increased in stage III)	Healthy controls	Stage III vs. control: 0.746 (95% CI 0.6347–0.8568); Stage III vs. stage II: 0.789 (95% CI 0.6407–0.9367); Stage III vs. stage IV: 0.738 (95% CI 0.6169–0.8593); *n* = 103	Prospective study	Disease-staging (stage III-specific)	
hsa-miR-22-5p	Serum	sEVs	Increased (specifically Increased in stage IV)	Healthy controls	Stage IV vs. control: 0.817 (95% CI 0.7023–0.9312); Stage IV vs. stage II: 0.773 (95% CI 0.5495–0.9959); Stage IV vs. stage III: 0.700 (95% CI 0.5713–0.8291); *n* = 103	Prospective study	Disease-staging (stage IV-specific)	
hsa-miR-151a-5p	Serum	sEVs	Increased (specifically Increased in stage IV)	Healthy controls	Stage IV vs. control: 0.741 (95% CI 0.6065–0.8744); Stage IV vs. stage II: 0.796 (95% CI 0.60–0.9909); Stage IV vs. stage III: 0.708 (95% CI 0.5812–0.8344); *n* = 103	Prospective study	Disease-staging (stage IV-specific)	
hsa-miR-24	Serum, CSF	EVs	Increased	Healthy controls	–	Secondary study	Diagnostic	(34)
hsa-miR-331-5p	CSF, Serum, Plasma	EVs	Increased	Healthy controls	–	Secondary study	Diagnostic	

α-syn, alpha-synuclein; ApoA1, apolipoprotein A1; AUC, area under the curve; CBS, corticobasal syndrome; CI, confidence interval; clu, clusterin; CSF, cerebrospinal fluid; DLB, dementia with Lewy bodies; EVs, extracellular vesicles; FTD, frontotemporal dementia; HC, healthy controls; hsa-miR, human microRNA; miR, microRNA; MSA, multiple system atrophy; n, number of participants/samples; NDEVs, neuron-derived extracellular vesicles; PD, Parkinson’s disease; PDD, Parkinson’s disease dementia; PSP, progressive supranuclear palsy; RBD, REM sleep behavior disorder; sEVs, small extracellular vesicles.

## 5 Extracellular vesicles as biomarkers in Huntington’s disease

Huntington’s disease (HD) is an autosomal dominant neurodegenerative disorder caused by a pathological CAG trinucleotide repeat expansion within the HTT gene. This mutation leads to the production and intracellular aggregation of mutant huntingtin (mHTT) protein, ultimately resulting in progressive neuronal dysfunction and cell death, particularly within the striatum and cerebral cortex (35). Although genetic testing identifies individuals carrying the pathogenic HTT mutation, it does not provide dynamic information on ongoing CNS pathology or disease progression, underscoring the need for fluid and imaging biomarkers—particularly in the premanifest stage (36). Consequently, there remains a critical need for biomarkers capable of reflecting early neurodegenerative alterations and disease state in HD. Given that extracellular vesicles (EVs) can cross the blood–brain barrier and carry proteins, lipids, and nucleic acids derived from their parental cells, peripheral EV-based liquid biopsy has emerged as a promising strategy for the early detection and assessment of HD-related neuropathological changes.

Brás et al. demonstrated that mHTT protein can be detected in plasma neuron-derived EVs (NDEVs) from patients with HD, supporting the feasibility of peripheral EV profiling for accessing disease-associated HTT in a minimally invasive manner. Given the established use of soluble CSF mHTT as a pharmacodynamic readout in HTT-lowering studies, plasma NDEV-associated mHTT may represent a potential candidate biomarker for pharmacodynamic assessment and therapeutic response monitoring in future studies (37). This finding supports the potential of plasma NDEV-associated mHTT as a candidate pharmacodynamic/response monitoring biomarker for HD. Pérez-González et al. reported significantly reduced levels of the ESCRT-associated protein Alix in sEVs isolated from postmortem striatum and cortex of HD patients, as well as in EVs secreted by HD fibroblasts. Alix levels in brain tissue also decreased with neuropathological severity (38). As an accessory component of the ESCRT-III machinery, Alix participates in multivesicular body (MVB) formation and vesicular cargo sorting prior to sEV release. Therefore, its reduction may indicate that intracellular mHTT aggregation interferes with EV biogenesis and cargo-loading pathways, thereby contributing to alterations in EV molecular composition (38, 39). Accordingly, current evidence suggests that Alix may be relevant to disease progression, although its diagnostic utility for HD remains to be established.

At the level of functional small non-coding RNAs, Herrero-Lorenzo et al. reported that plasma EV-derived tRF-Gly-GCC and miR-27a-3p levels were significantly decreased in individuals with premanifest HD (P-HD), whereas plasma neurofilament light chain (NfL) concentrations were significantly elevated relative to healthy controls (40). Importantly, a combined biomarker model incorporating tRF-Gly-GCC, miR-27a-3p, and plasma NfL demonstrated excellent discriminative performance for distinguishing P-HD individuals from healthy controls (AUC = 0.908, 95% CI = 0.82–0.99) (40). In addition, EV-derived miR-21-5p and miR-26a-5p levels were significantly associated with clinical and cognitive measures, including the Symbol Digit Modalities Test (SDMT) and the composite Unified Huntington’s Disease Rating Scale (cUHDRS) score (40). These findings suggest that EV-associated small RNAs may reflect early molecular alterations related to neuronal dysfunction and disease progression prior to overt clinical onset. Given that tRF-Gly-GCC and miR-27a-3p are small RNAs with emerging roles in neuronal function, while NfL is an established marker of axonal injury, their coordinated alterations in plasma EV and cell-free fractions may indicate the presence of early neurodegenerative changes during the premanifest stage.

## 6 Extracellular vesicles as biomarkers in stroke

Stroke is an acute central nervous system disorder broadly classified into ischemic and hemorrhagic subtypes, with contemporary clinical and basic research heavily focused on ischemic stroke. According to the Global Burden of Disease (GBD) 2023 study, there were approximately 13.2 million incident strokes worldwide in 2023, of which ischemic stroke accounted for 63.9% (approximately 8.4 million cases) (41). Given the associated high mortality and disability rates, early diagnosis of ischemic stroke is of paramount importance. However, current clinical workflows remain predominantly reliant on neuroimaging modalities such as CT and MRI, underscoring the vital clinical value of developing rapid, sensitive, and non-invasive liquid biopsy biomarkers (42). As direct executors of biological functions, altered proteomic profiles encapsulated within EVs may reflect pathological states associated with CNS injury. Lu et al. demonstrated that SIRT2 protein levels in serum-derived EVs from patients with acute ischemic stroke were markedly elevated and significantly associated with NIHSS scores, and showed a diagnostic performance for AIS (AUC = 0.838, 95% CI 0.775–0.902), suggesting its potential utility in clinical detection and disease severity assessment. However, SIRT2 did not independently predict 90-day functional outcome (mRS), so its prognostic value remains unestablished (43).

EV-associated non-coding RNAs exhibit considerable molecular heterogeneity and may provide multidimensional diagnostic information for distinct clinical needs. In the context of severity assessment and brain injury evaluation, Serum EV-associated miR-134 levels are significantly elevated in acute ischemic stroke patients and yield a diagnostic performance for discriminating AIS from non-stroke controls (AUC = 0.834). Its expression displays a positive correlation with both NIHSS scores (*r* = 0.6079, *p* = 6.4 × 10^−5^) and infarct volume (*r* = 0.7841, *p* = 4.3 × 10^−5^), suggesting it may serve as a potential molecular correlate of acute brain injury severity (44). For the determination of onset time windows and dynamic pathological staging, EV-associated non-coding RNAs may offer temporal clues. Wang et al. reported that plasma EV-associated miR-30a-5p is significantly elevated during the hyperacute phase of ischemic stroke (HIS, <6 h; AUC = 0.826, 95% CI 0.665–0.988), whereas miR-21-5p is upregulated during the subacute (SIS, 8–14 days; AUC = 0.714, 95% CI 0.570–0.859) and recovery phases (RIS, >14 days; AUC = 0.734, 95% CI 0.596–0.871). Notably, miR-21 is also elevated in glioma (see Section 7), indicating that it is not specific to stroke and may reflect shared pathological processes such as neuroinflammation or tissue remodeling. This stage-specific expression signature may provide a potential molecular framework for delineating onset time windows and optimizing thrombolysis, although the findings are limited by small sample sizes (especially HIS, *n* = 15) and lack of independent validation (45). Similarly, plasma sEV-associated miR-422a and miR-125b-2-3p exhibited phase-specific expression patterns in ischemic stroke. miR-422a was elevated in the acute phase but decreased markedly in the subacute phase, whereas miR-125b-2-3p was significantly reduced in the subacute phase relative to both controls and the acute phase, with no significant difference between the acute phase and controls. ROC analysis showed that sEV-associated miR-422a discriminated acute- from subacute-phase patients (AUC = 0.957, 95% CI 0.905–1.000), suggesting its potential as a candidate disease-staging biomarker (46).

Furthermore, compared to linear RNAs, EV-associated circRNAs exhibit high stability and brain enrichment due to their covalently closed loop structure. Jiang et al. identified five circRNAs (hsa_circ_0007290, hsa_circ_0049637, hsa_circ_0000607, hsa_circ_0004808, and hsa_circ_0000097) that were significantly upregulated in brain-derived EVs from plasma of acute ischemic stroke (AIS) patients compared to non-AIS controls. A five-circRNA diagnostic panel yielded a diagnostic performance (AUC = 0.921; *n* = 172 AIS, 67 controls), with 95.08% sensitivity and 80.65% specificity (47).

## 7 Extracellular vesicles as biomarkers in glioma

As the most prevalent primary malignant central nervous system malignancy, glioma is clinically stratified into low-grade and high-grade malignancies based on its degree of malignancy. Among these, glioblastoma (GBM) represents the most malignant, highly invasive subtype characterized by an exceedingly dismal prognosis (48). Currently, glioma diagnosis relies predominantly on neuroimaging modalities such as MRI; however, these techniques struggle to definitively differentiate among high-grade gliomas, low-grade gliomas, and brain metastases originating from extracranial tumors. Consequently, gold-standard confirmation still necessitates surgical biopsy, severely constraining early diagnostic intervention (49). Leveraging the established biological capacity of EVs to traverse the blood–brain barrier, their encapsulated molecular cargo naturally represents an ideal vehicle for glioma liquid biopsies. Manda et al. validated that EGFRvIII transcript in serum EVs serves as a noninvasive diagnostic biomarker for high-grade gliomas. Within a cohort of 96 high-grade glioma patients, the detection of EV-associated EGFRvIII yielded a sensitivity of 81.6%, a specificity of 79.3%, and an overall diagnostic accuracy of 80%, demonstrating moderate concordance with traditional histopathological profiling (*κ* = 0.548) (50).

Shi et al. observed that EV-associated miR-21 levels within the CSF of glioma patients were significantly elevated compared to non-tumor controls (traumatic brain injury patients) (*n* = 70 vs. *n* = 25), whereas no significant divergence was detectable in serum-derived EV fractions (*n* = 50 vs. *n* = 25). ROC curve analysis revealed that CSF EV miR-21 distinguished glioma from non-neoplastic conditions (AUC = 0.927, 95% CI 0.865–0.985), and high miR-21 levels were significantly associated with tumor recurrence patterns, including diffuse intracranial and spinal dissemination (*p* < 0.001). As noted in Section 6, miR-21 is also upregulated in the subacute phase of ischemic stroke, suggesting that it is not a glioma-specific biomarker. However, this was a single-center study without independent external validation. These findings highlight the distinct diagnostic efficacy inherent to different biofluid matrices, supporting the potential of CSF EV-associated miR-21 as a diagnostic and prognostic biomarker for glioma (51). In a separate study, Santangelo et al. found that the expression of miR-21, miR-222, and miR-124-3p in serum EVs of GBM patients was significantly higher than in healthy controls (*n* = 44 vs. *n* = 30), with combined detection delivering higher diagnostic efficacy (AUC = 0.87, 95% CI 0.7885–0.9524) compared with miR-21 alone (AUC = 0.84, 95% CI 0.7538–0.9371). It was also revealed that elevated miR-21 combined with normal miR-222 and miR-124-3p levels might indicate non-glial brain metastases rather than glioma. Furthermore, miR-21 expression was significantly higher in high-grade gliomas than in low-grade gliomas (AUC = 0.83, 95% CI 0.7395–0.9398), suggesting an association with tumor grade. These findings support the potential of serum EV-associated miRNAs as diagnostic, differential diagnostic, and disease-staging biomarkers for glioma (49). Beyond microRNAs, EV-associated circular RNAs (circRNAs)—favored for their structurally stable closed-loop conformation—show promise as diagnostic biomarkers for high-grade astrocytomas (HGAs). Li et al. screened a three-circRNA panel comprising hsa_circ_0075828 (upregulated), hsa_circ_0002976 (downregulated), and hsa_circ_0003828 (downregulated). An expression shift matching the designated direction in any two of these three candidates achieved 100% accuracy in discriminating HGA patients (*n* = 30) from healthy controls (*n* = 12) in this small single-center cohort. These findings support the potential of EV-associated circRNAs as diagnostic biomarkers for HGA, although independent validation in larger cohorts is needed (52).

## 8 Extracellular vesicles as biomarkers in multiple sclerosis

Multiple sclerosis (MS) is an autoimmune demyelinating disease of the CNS characterized by a highly heterogeneous clinical trajectory, manifesting primarily across distinct evolutionary subtypes including relapsing–remitting MS (RRMS) and progressive MS (PMS). Currently, the classification of MS disease activity and subtype stratification remain heavily reliant on clinical relapse history and magnetic resonance imaging (MRI) (53). Galazka et al. demonstrated that MOG levels within serum EVs are significantly elevated in patients during RRMS relapses and in secondary progressive MS (SPMS) cohorts compared to healthy controls, and correlate with Gd-enhancing lesions. This underscores the potential utility of EV-associated MOG as a candidate biomarker for active RRMS relapses (54).

At the transcriptomic level, dynamic changes in EV-associated miRNA profiles may offer potential liquid biopsy strategies for assessing disease activity and discriminating clinical phases. Regarding disease activity and relapse discrimination, transcriptomic profiling by Mycko et al. revealed that circulating EV-associated levels of miR-122-5p, miR-196b-5p, miR-301a-3p, and miR-532-5p are markedly suppressed in RRMS patients, reaching nadir levels during acute clinical relapses and active MRI lesion phases. Among these, a panel combining miR-122-5p and miR-196b-5p showed the best diagnostic performance, discriminating relapsing patients from healthy controls (AUC = 0.904) and acute relapses from clinical remission (AUC = 0.866; discovery *n* = 29, validation *n* = 95) (55). For subtype stratification, Ebrahimkhani et al. identified significantly differentially expressed serum sEV-associated miRNAs between RRMS and progressive MS phenotypes. In the discovery set, a three-miRNA combination (miR-223-3p, miR-485-3p, and miR-30b-5p) achieved 95% accuracy (Random Forest out-of-bag error 0.05) for distinguishing RRMS from progressive MS (56). Complementing these findings, multiple independent cohorts have sequentially identified miR-150-5p, Let-7 g-5p, miR-155, miR-18a-5p, and miR-145-5p as significantly dysregulated entities within the serum or cerebrospinal fluid (CSF) EV fractions of MS patients, expanding the candidate biomarker repository for advanced diagnostic development (57, 58).

## 9 Extracellular vesicles as biomarkers in amyotrophic lateral sclerosis

Amyotrophic lateral sclerosis (ALS) is a devastating, progressive neurodegenerative disorder characterized primarily by the selective degeneration of upper and lower motor neurons (59). This loss of motor function leads to progressive muscular weakness, muscle atrophy, and ultimately, fatal respiratory failure, typically occurring within 2 to 4 years of symptom onset (60). The histopathological hallmark of ALS involves the cytoplasmic mislocalization, hyperphosphorylation, proteolytic cleavage, and aggregation of TAR DNA-binding protein 43 (TDP-43), concomitantly paired with the depletion of physiological nuclear TDP-43 (61). Chatterjee et al. demonstrated that plasma sEV-associated TDP-43 discriminated ALS from healthy controls (AUC = 0.990, 95% CI 0.97–1.00) and from PSP (AUC = 0.99, 95% CI 0.98–1.00) in the DESCRIBE subcohort 2, and also distinguished ALS from bvFTD (AUC = 0.910, 95% CI 0.88–0.94). In an independent Sant Pau validation cohort, sEV TDP-43 again separated ALS from healthy controls (AUC = 0.940, 95% CI 0.89–0.99) and from PSP (AUC = 0.960, 95% CI 0.91–0.99), although discrimination from bvFTD was lower (AUC = 0.76, 95% CI 0.68–0.87). These findings support the potential utility of sEV-associated TDP-43 as a diagnostic and differential diagnostic biomarker for ALS (62).

Beyond TDP-43, proteomic profiling of plasma EV-associated proteins identified a marked upregulation of CORO1A in patients with ALS, with an approximately 5.3-fold increase relative to healthy controls. Subsequent validation by ELISA confirmed significantly elevated plasma CORO1A levels in 30 patients with ALS compared with 30 healthy controls. In a separate longitudinal subset, plasma CORO1A levels increased over 12 months in 8 of 10 patients, suggesting a potential association with disease progression. However, these findings were derived from a small discovery cohort (*n* = 3 per group) and a modest validation cohort (*n* = 30 per group), without reported ROC/AUC analyses or independent external validation. Moreover, the longitudinal analysis assessed total plasma CORO1A rather than EV-associated CORO1A. Further large-scale, independently validated studies are therefore needed to determine its diagnostic performance and clinical utility (63). Concurrently, dysregulation of serum EV-associated miRNAs has shown potential as a blood-based biomarker for ALS, although larger-scale validation is required. A meta-analysis and experimental validation identified miR-146a-5p as a significantly dysregulated EV-associated miRNA in ALS. In serum-derived EVs from patients with sporadic ALS (*n* = 22) and healthy controls (*n* = 18), miR-146a-5p levels were significantly elevated and showed diagnostic discrimination (AUC = 0.792; sensitivity, 100%; specificity, 50%). Moreover, miR-146a-5p levels were positively correlated with survival duration, suggesting a potential association with clinical outcome. Separately, miR-27a-3p was significantly downregulated in serum-derived EVs from ALS patients (*n* = 10) compared with healthy controls (*n* = 10), but no ROC/AUC analysis or independent validation was reported. However, both findings were derived from small cohorts, and neither has been independently validated, underscoring the need for larger, independently validated studies before their clinical utility can be established (64, 65).

## 10 Discussion

### 10.1 Diagnostic specificity and multidimensional biomarker strategies

The core value of extracellular vesicles (EVs) in the diagnosis of central nervous system (CNS) diseases is primarily reflected at two levels. First, EVs can carry disease-associated proteins, such as phosphorylated tau (p-tau) and α-syn, which may reflect pathological alterations within the CNS. Second, their encapsulated non-coding RNAs, including miRNAs and circRNAs, may reflect disease-associated molecular pathways and provide additional information for disease characterization and functional-state assessment. However, the clinical translation of EV biomarkers is challenged by substantial overlap in biomarker signals across different neurological diseases. For instance, miR-21 has been reported to be elevated in gliomas and is also significantly upregulated during the subacute phase of stroke; similarly, neurofilament light chain (NfL), a marker of axonal injury, is elevated across multiple neurological disorders, including Alzheimer’s disease (AD), amyotrophic lateral sclerosis (ALS), Huntington’s disease (HD), and stroke. These molecules may therefore reflect shared pathological processes, such as neuroinflammation or axonal degeneration, rather than a specific disease entity.

These observations suggest that future diagnostic strategies should move beyond the identification of a single disease-associated molecule toward multidimensional biomarker models. Disease-related proteins with relatively greater pathological specificity, such as p-tau, α-syn, or tumor-associated markers, could potentially be integrated with more broadly responsive indicators of neuronal injury or inflammation, such as NfL and selected RNA signatures. Such combinations may provide complementary information for distinguishing disease-specific pathological processes from common responses to neuronal injury. Future studies should therefore evaluate EV biomarker panels not only against healthy controls but also against clinically relevant neurological diseases with overlapping pathological features, thereby providing a more rigorous assessment of their differential diagnostic value.

### 10.2 Biofluid selection and attribution of EV cellular origin

The choice of biofluid source and techniques for tracing the central origin of EVs also merit careful consideration. Research indicates that the area under the curve (AUC) of cerebrospinal fluid (CSF) EV miR-21 in glioma patients reaches 0.927, whereas serum EV miR-21 shows no significant difference compared with controls. Conversely, in Parkinson’s disease (PD), hsa-miR-331-5p represents one of the few molecules consistently reported to be upregulated across CSF, serum, and plasma EVs. These findings highlight a potential trade-off between biological proximity and clinical accessibility: CSF EVs may be better suited for confirmatory biomarker discovery and mechanistic studies, whereas blood EVs offer greater feasibility for large-scale screening. Nevertheless, CNS-derived signals in blood may be diluted or confounded by abundant EVs from peripheral organs and blood cells, making reliable assignment of EV cellular origin particularly important.

Despite its widespread use, L1CAM-based immunocapture for neuron-derived EV (NDEV) enrichment remains controversial. Norman et al. reported that, in human plasma and CSF, L1CAM was predominantly detected in fractions corresponding to soluble proteins rather than EV-associated fractions after size-exclusion chromatography and density-gradient centrifugation, raising concerns regarding its suitability as a neuronal EV marker (66). Moreover, their antibody-based assay showed approximately threefold greater binding of recombinant α-syn to the anti-L1CAM antibody than to an IgG control, suggesting that nonspecific interactions between soluble proteins and capture antibodies may contribute to some previously reported L1CAM-associated signals (66). However, this interpretation is not universally supported. A subsequent single-EV study demonstrated that L1CAM-positive EVs co-expressed neuronal markers including β-III-tubulin, GAP43, and VAMP2; L1CAM-positive EVs carrying VAMP2 or β-III-tubulin accounted for 30–63%, compared with only 0.8–3.9% of L1CAM-negative EVs (67). These findings suggest that L1CAM may enrich a neuronal EV subpopulation, although L1CAM positivity alone may not definitively establish cellular origin.

Accordingly, alternative multi-marker strategies, including immunocapture based on GAP43/NLGN3 or ATP1A3, have been explored to improve neuronal EV enrichment, with neuronal origin further supported by orthogonal analyses of EV identity and neuronal cargo (68, 69). Beyond marker-based immunocapture, the recently proposed multifactor authentication framework advocates cross-validation of structural and molecular features rather than reliance on a single surface marker, potentially improving the specificity, reliability, reproducibility, and biological interpretability of EV subpopulation analysis (70). Spatial-EV-seq further extends this concept by enabling *in situ* EV capture, single-EV molecular profiling, and spatial mapping within native tissue microenvironments, thereby linking EV phenotypes to their anatomical and cellular context (71). Although these approaches have not yet been specifically established for circulating NDEVs, they represent promising directions for moving from single-marker enrichment toward multifactorial and spatially resolved attribution of neuronal EV origin.

### 10.3 Pre-analytical and analytical standardization

Another major challenge in the field is the lack of standardization across EV isolation and analytical workflows. Mainstream techniques, including ultracentrifugation, size-exclusion chromatography, and immunocapture, present inherent trade-offs between purity and yield, which can substantially affect cross-study comparability. In addition, pre-analytical variation represents a frequently underappreciated source of heterogeneity in blood-derived EV biomarker studies.

MISEV2023 emphasizes that donor characteristics and pre-analytical procedures—including diet, exercise, circadian rhythm, medication, blood collection, anticoagulant selection, handling time, centrifugation, and storage—should be systematically considered and reported. These factors can directly alter EV abundance or introduce non-EV particles into the analytical preparation. For example, overnight fasting is recommended to minimize postprandial chylomicrons, which may interfere with EV analysis (72). Platelet activation is another important source of variation because activated platelets can release large numbers of EVs; therefore, minimizing mechanical stimulation during venipuncture, standardizing the interval between blood collection and processing, and efficiently removing residual platelets are important for limiting platelet-derived EV contamination (73).

Hemolysis should likewise be assessed and reported because erythrocyte disruption releases intracellular components, including hemoglobin, that may confound downstream analyses (74). In addition, lipoproteins, including HDL, LDL, IDL, VLDL, and chylomicrons, overlap with EVs in size, density, or molecular composition, and some lipoprotein subclasses may outnumber blood EVs by several orders of magnitude (75). Consequently, apparent differences in EV particle abundance or molecular cargo may partly reflect variation in platelet contamination, hemolysis, or lipoprotein carryover rather than disease-associated EV biology.

Storage introduces another layer of variability. MISEV2023 notes that storage conditions can affect EV yield, cargo, functionality, and the ratio of individual particles to aggregates, whereas evidence regarding freeze–thaw effects remains heterogeneous (7). Therefore, storage temperature, duration, aliquoting, thawing procedures, and the number of freeze–thaw cycles should be standardized and explicitly reported, with repeated freeze–thawing minimized. Normalization procedures should likewise be predefined and consistently applied, with the selected denominator or reference metric justified according to the biological matrix and analytical platform.

Taken together, these considerations indicate that standardization should encompass the entire workflow rather than focusing exclusively on EV isolation. Transparent reporting of donor preparation, blood collection, anticoagulation, processing time, centrifugation, storage, freeze–thaw history, EV isolation, characterization, and normalization is essential for improving reproducibility and facilitating meaningful comparison across blood-derived EV biomarker studies.

### 10.4 Clinical validation and translational requirements

An important consideration in evaluating the clinical utility of EV-derived biomarkers is whether their diagnostic or prognostic associations remain informative after accounting for clinically relevant covariates. EV measurements may be influenced by demographic characteristics, disease duration and severity, treatment exposure, comorbidities, vascular risk factors, and systemic inflammatory states. Importantly, the extent of covariate adjustment has varied considerably across existing EV studies. Some studies have incorporated only basic demographic variables, whereas others have additionally considered disease duration, clinical severity, or treatment exposure. For example, in Parkinson’s disease, NDEV-associated oligomeric α-syn remained significantly associated with specific non-motor symptom domains after adjustment for age, sex, motor impairment, disease duration, and levodopa equivalent daily dose, with significant associations for perceptual problems/hallucinations (ρ = 0.285, *p* = 0.01), attention/memory (ρ = 0.293, *p* = 0.01), and miscellaneous symptoms (ρ = 0.355, *p* < 0.01). These associations were further supported by multivariable regression analyses incorporating the same covariates (76). Similarly, in glioblastoma, an EV-associated lncRNA signature remained independently associated with overall survival after multivariable adjustment for age, pharmacotherapy, radiotherapy, IDH mutation, and MGMT promoter status (*p* = 1.33 × 10^−4^), supporting its independent prognostic value beyond these clinicopathological factors (77). In amyotrophic lateral sclerosis, plasma astrocyte-derived EV pTDP-43/CD81 retained diagnostic information after adjustment for age and sex, yielding a mean AUC of 0.89 (95% CI, 0.75–0.99), with 87% sensitivity and 89% specificity in logistic regression models (78).

However, covariate adjustment does not establish the generalizability of an EV biomarker model. Several studies have reported exceptionally high diagnostic performance, with AUCs approaching 0.96–1.00, based on relatively small case–control cohorts. For example, a three-circRNA EV-associated signature was reported to achieve 100% diagnostic accuracy for high-grade glioma (52). Such findings should be interpreted cautiously because high performance estimates from small and highly selected cohorts may be susceptible to overfitting and may not be reproducible in independent populations. Thus, evidence of an association after covariate adjustment and evidence of reproducibility in independent populations represent complementary aspects of clinical validation. Although partial covariate adjustment provides evidence for the potential independent value of EV biomarkers, comprehensive adjustment for the full range of potentially influential factors—including comorbidities, vascular risk factors, medication exposure, and systemic inflammatory status—remains uncommon. Independent external validation, preferably in prospective multicenter cohorts, is therefore essential to determine whether the observed diagnostic or prognostic performance can be generalized beyond the original study population.

A systematic review of 471 EV-related clinical trials showed that EVs have already entered substantial clinical evaluation, with 47.4% of trials assessing diagnostic applications and 33.3% evaluating companion diagnostics. However, only 11% explicitly investigated EV subpopulations, and reporting of EV isolation and characterization procedures remained incomplete (79). Thus, the increasing volume of clinical EV research has not yet been matched by equivalent maturation in EV subtype attribution and analytical standardization, highlighting important barriers to clinical translation. A more advanced example is the urinary EV-based ExoDx Prostate (IntelliScore) assay, which progressed through multiple prospective validation studies before receiving FDA Breakthrough Device Designation and CE-IVD certification (80). Although this assay represents a non-CNS application, it illustrates the level of prospective validation and regulatory development required for EV-based diagnostics to progress toward clinical implementation. In contrast, CNS EV biomarkers have not yet reached a comparable level of independent clinical validation or regulatory maturity.

For CNS applications, future studies should therefore adopt a stepwise translational framework incorporating standardized pre-analytical and analytical procedures, predefined quality criteria, appropriate reference materials, clinically relevant covariate adjustment, independent external validation, and prospective multicenter evaluation. For diagnostic applications, the intended clinical use should also be defined early to guide subsequent regulatory assessment, including applicable FDA requirements in the United States and EU IVDR requirements in Europe. Ultimately, high discriminatory performance in exploratory cohorts should be regarded as an initial biomarker signal rather than sufficient evidence of clinical readiness; reproducibility, external validity, clinical utility, and regulatory fitness will determine whether CNS EV biomarkers can progress from promising candidates to clinically deployable diagnostic assays (79, 80).

To facilitate clinical translation, future studies should adopt a stepwise and standardized framework. Specifically, MISEV2023 reporting standards should be followed, EV isolation and characterization protocols harmonized, and pre-analytical procedures and normalization strategies standardized. Candidate biomarkers should be adjusted for clinically relevant covariates, including age, sex, medication, comorbidities, vascular risk factors, and inflammatory status, and validated in independent external cohorts, preferably through prospective multicenter studies with pre-registered designs. Multidimensional biomarker panels should also be evaluated against clinically relevant disease controls, and the intended clinical use should be defined early to guide regulatory assessment.

## 11 Conclusion

In summary, the protein and non-coding RNA cargoes encapsulated within extracellular vesicles exhibit characteristic expression profile alterations across various central nervous system (CNS) diseases, including AD, PD, stroke, and glioma. Leveraging their inherent biostability, capacity to penetrate the blood–brain barrier (BBB) via facilitated biomembrane fusion, and tissue-of-origin enrichability driven by specific surface markers (such as L1CAM), this novel liquid biopsy paradigm addresses a fundamental limitation of traditional peripheral blood diagnostics: the inability to access central pathological information. Consequently, this approach demonstrates robust clinical diagnostic value in early prediction, precise molecular subtyping, and the longitudinal tracking of disease progression.

In summary, extracellular vesicles (EVs) carry disease-associated proteins and non-coding RNAs that reflect diverse pathological processes across central nervous system (CNS) diseases, including AD, PD, stroke, glioma, MS, HD, and ALS. Their molecular cargoes and potential for cell-of-origin enrichment provide a promising basis for minimally invasive liquid biopsy approaches to the diagnosis and differential diagnosis of CNS diseases. In particular, EV-associated pathological proteins and regulatory RNAs may complement conventional clinical and imaging measures by providing molecular information relevant to early disease detection and differential diagnosis. However, the diagnostic value of EV biomarkers remains constrained by substantial overlap in molecular signals across CNS diseases, uncertainty regarding cellular origin, pre-analytical and analytical variability, and limited independent validation. Thus, high diagnostic performance reported in individual studies should not be considered sufficient evidence of clinical readiness without demonstrating reproducibility and clinical relevance in independent populations.

From a translational perspective, EV-based diagnostic development will require a clear definition of the intended clinical use, target population, and clinical decision that the assay is designed to support. Candidate biomarkers should progress from analytical validation and clinical performance assessment toward demonstration of clinical utility in appropriately designed patient cohorts. Once sufficient evidence has been established, the intended use and risk profile of the diagnostic assay will also determine the applicable regulatory pathway, including relevant FDA requirements in the United States and the EU *In Vitro* Diagnostic Medical Devices Regulation (IVDR) framework in Europe. Regulatory considerations should therefore be incorporated early in assay development, particularly with respect to analytical performance, manufacturing consistency, quality control, and evidence supporting clinical performance and clinical utility.

Ultimately, the translation of CNS EV biomarkers into clinical diagnostics will depend on whether their molecular signals can be reproduced across laboratories and patient populations and shown to provide clinically meaningful information beyond established diagnostic approaches. Establishing this evidence base will be essential for moving EV-based biomarkers from promising research candidates toward clinically deployable diagnostic assays.
